# Case Report: Littoral cell angioma of spleen

**DOI:** 10.4103/0971-3026.54886

**Published:** 2009-08

**Authors:** Netra Rana, Zhang Ming, Ma Shao Hui, Yan Bin

**Affiliations:** Department of Diagnostic and Medical Imaging, First Affiliated Hospital of Xian Jiao Tong University, Xian 710061, Shaanxi Province, P.R. China

**Keywords:** Immunohistochemistry, littoral cell angioma, malignancy

## Abstract

Littoral cell angioma is a rare primary vascular neoplasm of the spleen, composed of littoral cells that line the splenic sinuses of the red pulp. It was thought to be a benign, incidental lesion. However, many recent reports have described it to be a malignant lesion with congenital and immunological associations. The definitive diagnosis can only be made after histology and immunohistochemistry studies.

## Introduction

Littoral cell angioma (LCA) is a rare primary vascular neoplasm of the spleen, first described by Falk *et al*.[1] in 1991. It is thought to arise from the littoral cells that normally line the splenic sinuses of the red pulp. Littoral cell angioma was originally believed to be benign but many recent reports have suggested that it is a malignant lesion.[2–4] In this article, we would like to report two incidentally encountered cases of this rare splenic vascular neoplasm.

## Case Reports

### Case 1

**Figure 1 (A-D):**
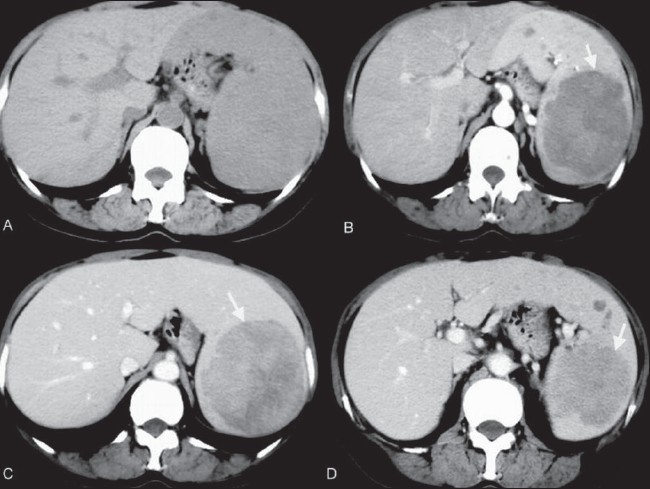
LCA of spleen. Case 1. CT scan. An axial nonenhanced image (A) shows no splenic mass. The arterial (B) and portal venous (C) images show a large, solitary, low-density lesion (arrow), the mass (arrow) becoming isodense to surrounding enhancing normal splenic tissue on the delayed phase image (D)

**Figure 2 (A-D):**
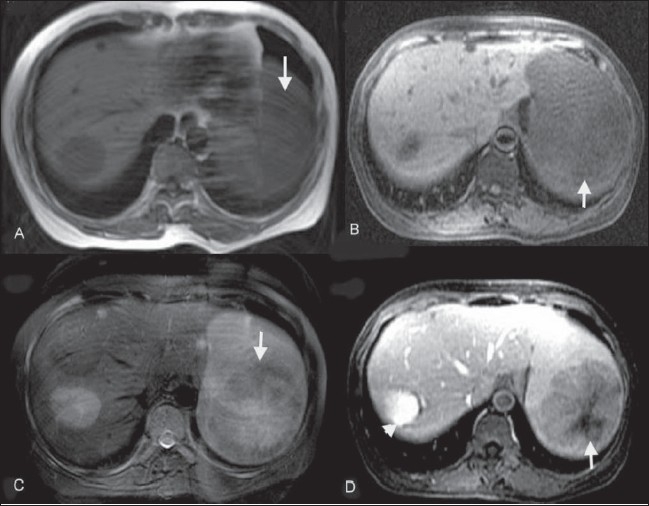
LCA of spleen. Case 1. MRI. Axial spin-echo T1W (A), axial gradient-echo T1W (B) and axial fast spin-echo T2W (C) images show a hypointense splenic mass (arrows). A contrast-enhanced image (D) shows heterogeneous enhancement (arrow). An incidental hemangioma is seen in the right lobe of the liver (arrowhead in D)

A 56-year-old Chinese woman presented with dizziness and tightness of the chest. She had been diagnosed with a thyroid adenoma with cystic degeneration 20 years ago and had persistent complaints of fatigue for the last 2 years. On physical examination, she was found to have hepatosplenomegaly. Blood examination showed moderate anemia; liver and renal function tests were normal. CT scan and MRI were performed. No splenic mass was seen on nonenhanced CT; [Figure 1A] however, a large, solitary, low-density lesion appeared in the early portal venous phase of the contrast-enhanced CT [Figure 1B and C]; this mass was isodense to the surrounding, enhancing normal splenic tissue on the delayed phase [Figure 1D]. MRI showed the mass to be hypointense on both T1W and T2W images [Figure 2A and C]. On the T1W image, another low-intensity solitary mass was seen in the right lobe of the liver [Figure 2A]; it was hyperintense on T2W images [Figure 2C], and was later diagnosed to be a cavernous angioma of the liver. Splenectomy was performed under general anesthesia. At surgery, splenomegaly was evident and a mass measuring about 10 cm in diameter was seen to be present. On gross pathological examination, a focal solitary mass measuring 9 × 9 × 7.5 cm with clear margins was found. Microscopically, the lesion was composed of anastomosing vascular channels with focal, projecting, papillary fronds and multiple cystic spaces exhibiting erythrophagocytosis. These spaces were lined with tall endothelial cells that were positive for endothelial and histiocytic markers CD31, CD68 and vimentin (VM), but negative for CD34 and CK, on immunohistochemical (IHC) examination. On the basis of these findings, LCA of the spleen was diagnosed.

### Case 2

**Figure 3 (A, B):**
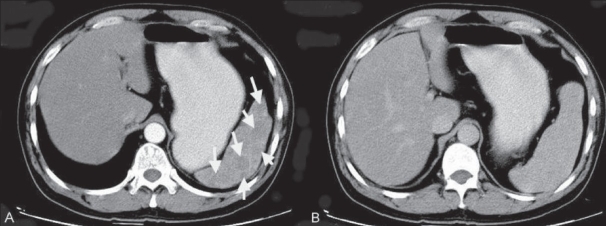
LCA of spleen. Case 2. A 47-year-old Chinese man with multiple splenic lesions. The arterial phase contrast-enhanced CT scan image shows multiple low-attenuation lesions (arrows). The delayed CT scan image (B) shows that the lesions have now become isodense to the adjacent splenic tissue

A 47-year-old Chinese male was found to have multiple splenic lesions. On B-mode USG, hemangioma was suspected and CT scan showed multiple lesions of the spleen [Figure 3]. The patient had a 2-year history of hypertension and had had pulmonary tuberculosis 25 years ago. After surgery, the microscopic and histopathological findings suggested that the splenic lesions were composed of variably sized lacunar cavities that were filled with red blood cells; the cavities were lined by a single layer of cuboidal or columnar cells. Hemosiderin and some foam cells were present. No tumor cells were found. The adjacent normal splenic tissue was compressed by the lesion. On IHC studies, the lesions were positive for CD31, CD68 and factor VIII and negative for CD34. The final diagnosis was LCA of the spleen.

## Discussion

LCA is usually asymptomatic (>55%) and is only discovered incidentally. It has no predilection for any particular age-group or sex; cases have been reported over the age range of 1–77 years (median age: 50 years) and in both sexes (female: male ratio = 5 : 3)[4,5]. However, according to Priego *et al.*[2] females are predominantly affected. Clinically, splenomegaly is almost always present; abdominal pain, pyrexia of unknown origin, symptoms of hypersplenism (thrombocytopenia and anemia) and portal hypertension may occasionally be seen.[1,6] Recent reports describe LCA as being associated with neoplasms of the colon, kidney, pancreas, lung and ovary.[2,3,4] Associations with leiomyosarcoma, melanoma and lymphoma have also been reported. In view of these findings, visceral neoplasm should be ruled out in all LCA patients.[2] Bi *et al*.[7] have noted that 17% of the cases of LCA were associated with immunological or congenital disorders such as Crohn's disease, Wiskott-Aldrich syndrome, Epstein syndrome, lymphocytic colitis, ankylosing spondylitis, Gaucher's disease, myelodysplastic syndrome, chronic glomerulonephritis, or aplastic anemia. Definitive diagnosis is possible only postoperatively, through histopathology and immunohistochemical studies.[3,4,7] LCA has the features of both endothelial and histiocytic differentiation, being positive for factor VIII, CD31, CD34, CD68, CD21, Mac-387, Ham-56, vimentin and lysozyme. Morphological findings include the presence of anastomosing vascular channels lined with tall endothelial cells and focal papillary fronds, with normal splenic sinuses at the periphery of the lesion.[1] Percutaneous fine-needle aspiration biopsy (FNAB) has been used successfully for the preoperative diagnosis of LCA and has been found to be a safe procedure with few complications; however, use of LCA is still controversial.[2,8]

On unenhanced CT scan, LCA masses are only rarely visible [Figure 1A]; however, on early-phase contrast-enhanced CT (during the portal venous phase), they appear as multiple low-attenuation lesions [Figure 3A], ranging in size from 0.2 to 6.0 cm. On delayed images, they are isodense with the surrounding enhancing splenic tissue [Figure 3B]. These CT findings correlate well with the gross appearance and histological findings. On USG, the features are less well-defined: there is a mottled echotexture, with multiple or focal hyperechoic diffuse, heterogeneous masses. On MRI, the splenic mass(s) typically appear hypointense on both T1W and T2W images [Figure 2B and C] due to the presence of hemosiderin in the lesions, a result of hemophagocytosis by neoplastic cells.[4,9,10] Radiological findings are rarely sufficient for making a definitive diagnosis because many other splenic neoplasms can mimic LCA. Hemangioma, lymphangioma, hamartoma, hemangiopericytoma, hemangioendothelioma and angiosarcoma; malignant processes such as metastases, lymphoma and Kaposi sarcoma; and infectious processes such as those due to Pneumocystis and Mycobacterium should always be kept in mind as differential diagnoses of LCA.[10]

In conclusion, LCA, is a rare vascular benign tumor of the spleen that may portend malignancy[2–4] and may also be associated with immunological and congenital disorders.[5] The treatment of choice for LCA is laparoscopic surgery with splenectomy. The imaging features of many other splenic neoplasms may mimic those of LCA but, in such cases, diagnostic signs and symptoms are usually present. If splenic mass(s) and nodule(s) is/are incidentally identified on imaging and the patient has no associated signs or symptoms, then LCA should be suspected.
